# A Genetically Modified Tobacco Mosaic Virus that can Produce Gold Nanoparticles from a Metal Salt Precursor

**DOI:** 10.3389/fpls.2015.00984

**Published:** 2015-11-10

**Authors:** Andrew J. Love, Valentine V. Makarov, Olga V. Sinitsyna, Jane Shaw, Igor V. Yaminsky, Natalia O. Kalinina, Michael E. Taliansky

**Affiliations:** ^1^Cell and Molecular Sciences, The James Hutton InstituteDundee, UK; ^2^A.N. Belozersky Institute of Physico-Chemical Biology, Lomonosov Moscow State UniversityMoscow, Russia; ^3^Chemical Faculty, Lomonosov Moscow State UniversityMoscow, Russia; ^4^Physical Faculty, Lomonosov Moscow State UniversityMoscow, Russia

**Keywords:** plant virus, green synthesis, metal nanoparticles, metal ion reduction, functional peptides

## Abstract

We genetically modified tobacco mosaic virus (TMV) to surface display a characterized peptide with potent metal ion binding and reducing capacity (MBP TMV), and demonstrate that unlike wild type TMV, this construct can lead to the formation of discrete 10–40 nm gold nanoparticles when mixed with 3 mM potassium tetrachloroaurate. Using a variety of analytical physicochemical approaches it was found that these nanoparticles were crystalline in nature and stable. Given that the MBP TMV can produce metal nanomaterials in the absence of chemical reductants, it may have utility in the green production of metal nanomaterials.

## Introduction

In spite of the emerging importance of nanoparticles, there are a multitude of caveats associated with their synthesis which may restrict their marketability or technological application. Nanoparticles are commonly synthesized by reducing metal salts in solution into small assemblages of metal atoms via the use of hazardous chemicals such as sodium borohydride, tetrakishydroxymethylphosphonium chloride (THPC), poly-*N*-vinyl pyrrolidone (PVP), and hydroxylamine (Narayanan and Sakthivel, 2011). In contrast, physical methods of nanoparticle production involve vapourization of metals and their subsequent deposition and coalescence onto various substrates, leading to formation of nanosized structures (Tsuji et al., 2003; Kundu et al., 2007; Okitsu et al., 2007). Although these synthesis approaches have been used successfully to produce well-defined nanoparticles, they may be hazardous and potentially dangerous to the environment (Narayanan and Sakthivel, 2011). Moreover, there is a possibility that some of the chemicals used to synthesize the nanostructures may adsorb onto the nanoparticle surface, rendering it toxic and unable to be used in some applications that require interaction with living organisms; such as in the medical field (Gan and Li, 2012). Therefore, there is a clear need for cost-effective, non-hazardous, and eco-friendly methods of nanoparticle production (Raveendran et al., 2003; Sharma et al., 2009; Narayanan and Sakthivel, 2011).

Recently the metal ion reducing and binding capacities of proteins, peptides, and amino acids have been exploited for the non-hazardous production of nanoparticles from metal salts; reactions which can be carried out at room temperature and neutral pH, with reduced environmental costs and increased biocompatibility (Tan et al., 2010). Amino acids can be used for nanoparticle synthesis as they have different side chains which can participate to different extents in both metal ion sequestration and/or reduction of metal ions (Naik et al., 2002; Wangoo et al., 2008; Tan et al., 2010). For example, metal ion binding can occur via N-terminal main-chain amino and C-terminal carbonyl groups, or through side chains such as the ring nitrogen atom of histidine, or the carboxylate groups of aspartic and glutamic acid. Metal ion reduction may be facilitated by hydroxyl groups in tyrosine and carboxyl groups in asparagine and glutamine, and indole groups on tryptophan. Tan et al. (2010) investigated how various juxtaposed metal ion binding and metal ion reducing amino acid sequences can influence this process (Tan et al., 2010). They found that in order to adequately form nanoparticles from metal ions, the peptide sequence has to have potent reductant capacity and have the potential to attract metal ions to the reduction sites without excessive chelation. Moreover, they showed that the sequence configuration could control the size, morphology and numbers of gold nanoparticles formed in reactions from chloroauric acid. For example the peptide GASLWWSEKL, rapidly reduces metal ions into large numbers of <10 nm nanoparticles, whereas swapping the N- and C-terminal regions of the peptide (SEKLWWGASL) leads to a slower reaction that results in the production of gold 40 nm spherical nanoparticles and nanotriangles.

While such peptides have excellent potential in nanoparticle synthesis, there are still cost and scalability issues with regard to their large scale production for use in commercial applications. Although peptides can be produced using yeast and bacterial expression systems, plant expression systems have emerged as a cheaper, safer, faster, and more scalable platform for protein and peptide production (Rybicki, 2010). For example, large scale production of recombinant proteins in plants is 10–50- fold cheaper than those produced using bacteria (Kusnadi et al., 1997). In this paper we have exploited the use of plants as factories to produce novel virus-based structures that are surface decorated with peptides which can promote the formation of metal nanoparticles. Given that it was previously shown by Tan et al. (2010) that the SEKLWWGASL peptide can induce the formation of 10–100 nm metal nanoparticles (which are the sizes that are most commonly used in nanotechnology), we used genetic approaches to introduce this peptide into a surface exposed loop of the coat protein of tobacco mosaic virus (TMV). TMV is a rod-shaped helical plant virus with a length of 300 nm, a diameter of 18 nm, and a central channel 4 nm in width (Clare and Orlova, 2010). It has a plus-sense single stranded RNA genome of 6395 nucleotides encapsidated by 2130 identical coat protein subunits of 17.5 kDa each; thus each modified TMV particle contains 2130 surface exposed metal nanoparticle forming peptides. The modified virus which was produced and isolated from the host plant *Nicotiana benthamiana* was shown to elicit the formation of gold nanoparticles when mixed with a K(AuCl_4_) precursor. This paper describes a method for production of crystalline, stable free gold nanoparticles using a virus but without the addition of exogenous reductants.

## Materials and Methods

### Introduction of the Metal Binding and Reducing Peptide Sequence into C-terminal Surface Exposed Loops of TMV Coat Protein

Plasmid pSNC004 containing a full-length TMV U1 genome under the control of a T7 promoter and modified in its coat protein sequence at nucleotides 193–198 and 208–213 to contain NgoMIV and BstZ17I sites, respectively, was provided by Dr Sean Chapman (The James Hutton Institute, Invergowrie, Dundee, DD2 5DA, UK). The plasmid was digested with these restriction enzymes and treated with Calf Intestinal Phosphatase (according to the enzyme manufacturers protocols; New England Biolabs, 240 County Road, Ipswich, MA, USA) prior to electrophoresis on 1xTBE 1% agarose gels and purification using a Qiagen gel extraction kit (Qiagen; Lloyd Street North, Manchester, M15 6SH, UK). The digested plasmid was then ligated to the MBP primers discussed below.

The MBP primers (Au2F and Au2R) below correspond to the SEKLWWGASL sequence which is flanked with incomplete restriction sites to enable downstream ligation to the digested plasmid (the incomplete recognition sequence of NgoMIV is underlined in bold, whereas that of BstZ17I is italicized in bold). These primers which were synthesized by Eurofins (Anzinger Str. 7a, 85560, Ebersberg, Germany) were modified to contain 5′ phosphates.

Au2F 5′ **CCGG**CTCTGAAAAGCTTTGGTGGGGAGCTTCTCT***GTA*** 3′

Au2R 5′ ***TAC***AAGAGAAGCTCCCCACCAAAGCTTTTCAGA**G** 3′

Primers were annealed by mixing 2 μM of each primer in a 10 mM Tris pH8, 50 mM NaCl, and 1 mM EDTA reaction mix. Reactions were placed in a 96°C heating block for 2 min, after which the heating block was turned off and left until the temperature returned to room temperature. The annealed primer mix was then ligated to the BstZ17I/NgoMIV digested and CIP treated plasmid according to the New England Biolabs (NEB; 240 County Road, Ipswich, MA, USA) T4 DNA ligase protocols. Two microliters of each ligation was mixed with 50 μl of electrocompetent XL1 blue *Escherichia coli* and electroporated according to the protocols of Sambrook and Russell (2001) prior to plating out on 100 μg/ml ampicillin LB plates. The plates were incubated at 37°C in a static incubator in order to permit growth of ampicillin resistant colonies. Colonies were selected and used to inoculate 100 μg/ml ampicillin LB liquid media, which were then incubated at 37°C in a 200 rpm shaker overnight. The turbid cultures were centrifuged at 4000 *g* to pellet the bacteria prior to plasmid extraction using a Qiagen Miniprep Kit (Qiagen; Skelton House, Manchester, UK). The isolated plasmids were sent for sequencing at the James Hutton Institute sequencing service (The James Hutton Institute, Invergowrie, Dundee, DD2 5DA, UK) in order to confirm successful integration of the sequence of interest. Those clones with the correct sequence were subsequently used for generation of infectious TMV transcripts.

### Amplification and Isolation of Modified TMV

Prior to *in vitro* transcription, the plasmids were linearized by KpnI digestion and then electrophoresed and purified as described above. The concentration of the digested plasmids was quantitated spectrophotometrically, and 750 ng of each plasmid was then used in a T7 *in vitro* transcription reaction according to the manufacturer’s protocols (Ambion T7 mMessage mMachine; Life Technologies, 3175 Staley Road, Grand Island, NY 14072, USA). Reactions were diluted 1:50 and abrasive carborundum powder was added, and 10 μl of this solution was rubbed onto the uppermost emergent leaves of *N. benthamiana* plants at the five leaf stage of development. Plants were grown under 16 h daylength at 23°C. Progression of symptoms was observed over a 16 days period after which material was harvested for virus isolation. The material was ground to a powder using liquid nitrogen and mortar and pestles prior to addition of two parts (w/v) 4°C 0.1 M sodium phosphate buffer pH 7.8, 20 mM EDTA (BGH Laboratory Supplies, England, UK), 0.1% β-mercapthoethanol (Sigma–Aldrich, UK). After incubation at 4°C for 1 h, the solution was centrifuged at 10,000 *g* for 20 min at 4°C. Six parts supernatant was mixed with one part chloroform and then centrifuged as before. PEG (Sigma–Aldrich, UK) and NaCl (Sigma–Aldrich, UK) were then added to the supernatant at a final concentration of 2 and 1%, respectively, and precipitation was performed overnight at 4°C. After another centrifugation step the pellet was resuspended in 25 mM Tris-HCl buffer (pH 7.8). The virus suspension was purified by a low speed 4°C centrifugation at 2,300 *g* for 5 min, followed by supernatant ultracentrifugation at 4°C on a 20% sucrose (BDH AnalaR, England, UK) cushion for 2 h at 32,000 rpm in a SW 41 Beckman Coulter Optima L-80 XP rotor (Beckman Coulter, Harbor Boulevard, CA, USA). The pellet was dissolved in 0.01 M Tris-HCl buffer (pH 7.8) and a second round of ultracentrifugation was performed as before but without a sucrose cushion. The virus pellet was resuspended in ultrapure water (Qiagen, UK) and stored on ice at 4°C.

Tobacco mosaic virus particle yield was quantitated spectrophotometrically using A260, A280, and A320 measurements according to Chapman (1998).

### Western Blot Analysis of Virus

Non-infected, wild type (WT) TMV and MBP TMV infected *N. benthamiana* leaves were harvested for western blotting 16 days after inoculation. Approximately 30 mg of leaf material was ground in liquid nitrogen, combined with 100 μl of 2X Laemmli loading buffer and then boiled for 5 min to denature the proteins. After centrifugation at 16,000 rpm, 20 μl was loaded onto 15% SDS-PAGE gels, along with 15 μl of Novex prestained ladder (Invitrogen, Paisley, UK). Gels were electroblotted onto Immobilon-P membrane (Millipore, Watford, UK). The Immobilon-P membrane was blocked by incubating in 1X PBS, 1% BSA and 0.05% Tween, for 1 h. Anti-TMV antibodies raised in rabbits were added to the membranes in blocking solution at a 1/10000 dilution and incubated with shaking at room temperature for 1 h. After washing, an anti-rabbit IgG alkaline phosphatase conjugate was added to the blots at a concentration of 1/1000 in blocking buffer (A8025-1ML; Sigma, Dorset, UK). After incubation at room temperature under shaking conditions the blots were washed and covered with BCIP/NBT (B1911; Sigma, Dorset, UK). The blots were left to develop for 10 min, after which banding was visible. The developed blots were scanned and saved as jpeg images.

### Characterization of WT and MBP TMV using Transmission Electron Microscopy (TEM), Atomic Force Microscopy (AFM), Dynamic Light Scattering (DLS) and Zeta Potential Measurements

#### Transmission Electron Microscopy (TEM) on Virus Samples and Immunotrapping

Viruses diluted in sterile filtered distilled water were adsorbed onto carbon-coated copper grids which were examined in a JEOL 1400 transmission electron microscope (TEM) at 80 kV.

For immunotrapping, TMV antisera (raised in rabbits) was diluted 1/1000 in 0.7 M Sorenson’s phosphate buffer and 30 μl was applied to carbon coated grids and incubated for 60 min at 37°C. These grids were subsequently washed twice in Sorenson’s by incubating them each time for 10 min on a rotating table. Ground plant material was mixed with buffer to give 1 in 4 and 1 in 40 dilutions (w/v) and filtered through miracloth, prior to overlaying 20 μl of the sample with the antisera coated grids. These were incubated overnight at 4°C prior to washing twice with 50 μl sterile water, treating with 2% uranyl acetate and then air drying before viewing on the TEM.

#### AFM Analysis of Viruses

Purified virus was adsorbed onto the surface of freshly cleaved mica for 1 min, after which the solution was carefully removed with filter paper. This substrate was immediately placed on to a drop of double-distilled water (this procedure was repeated twice), and the surface was dried under air flow. This sample preparation method was used to eliminate any remaining salts and minimize any risk of aggregation during drying. AFM analysis was performed on these samples using a Nanoscope IIIA microscope (Digital Instruments, USA) operating in tapping mode with a typical scan rate of 1 Hz. The measurements were performed in air in tapping mode using sharp silicon cantilevers (NT-MDT, Russia) with a guaranteed tip radius of 10 nm.

#### DLS and Zeta Potential Measurements on Virus Samples

Virus preparations were analyzed using DLS and Zeta potential techniques. Measurements were carried out using a Zetasizer Nano ZS device (Malvern Instruments Ltd., Great Britain) with a He-Ne laser (633 nm, 10 mW) as a light source. Sample temperatures were maintained at 0.1°C using a Peltier thermostatting system. Light scattering was measured at an angle of 173°, with subsequent detection and processing of autocorrelation functions being carried out using Dispersion Technology Software (DTS) version 5.10. Polystyrene cells with 10 mm optical paths were used for the DLS experiments, where sample volumes were 1 ml and the concentration of virus preparation was in the range of 0.15–0.3 mg/ml. Zeta potential measurements were carried out using the same sample volume and virus concentration range specified above, except that the samples were decanted into folded capillary cells (Malvern Instruments Ltd., Great Britain) for analysis.

### Production of Gold Nanoparticles using Viruses

In a 200 μl reaction, 0.1 mg of purified TMV (WT or MBP TMV) was combined with 3 mM of potassium tetrachloroaurate (prepared in filtered sterile distilled water) and shaken at 100 rpm at 20°C.

#### Analysis of Gold Nanoparticles using Physicochemical Methods

Gold nanoparticles were characterized using TEM, AFM, absorbance spectroscopy, selected area electron diffraction analysis (SAED), and Dynamic light scattering (DLS). A LEO912 AB OMEGA TEM operating at 100 kV was used in tandem for image capture and SAED. The nanoparticle samples were diluted 1:10 or 1:100 and pipetted onto formvar coated copper grids, which were then air dried prior to insertion into the microscope. AFM measurements were carried out in the same way as for the virus samples.

For DLS measurements, samples of gold nanoparticles were loaded into 1 cm cells of the Zetasizer Nano ZS (Malvern Instruments, UK), and measurements were obtained using the He-Ne laser (633 nm). Curves were fitted using DTS version 5.10.

UV-visible measurements in the 300–750 nm range were measured in cells with an optical path of 1 cm, using a Beckman DU640 spectrophotometer. The spectra of the viruses were used as a baseline and subtracted from the spectra of a mixture of metal salt/acid and viruses.

For XRD, precipitated gold nanoparticles were measured using a DRON-3M X-ray diffractometer with a CuKα X-ray source (aaa = 1.5418 Å) operated at 30 kV and 20 mA. Powder X-ray diffraction data were measured in the 2Θ range from 35 to 45°.

EDS measurements were obtained using a LEO 1430-vp (Carl Zeiss) field emission scanning electron microscope equipped with an Oxford energy dispersive detector INCA. The process of sample preparation consisted of drying a 10 μl drop on a silicon substrate. EDX spectra were measured for separate particles to confirm their chemical composition.

## Results

### Production of Viruses and their Characterization

*Nicotiana benthamiana* plants inoculated with WT TMV developed the classical symptoms of stunting, leaf malformation and chlorophyll loss at 16 days post inoculation (dpi). In contrast, slightly more severe stunting and malformation was observed in plants inoculated with the TMV modified to display the SEKLWWGASL metal binding peptide (MBP TMV) in the surface exposed loop of the TMV coat protein (the peptide replaced the 66–75 aa region of the coat protein). At 16 dpi, plants were harvested and some material was used for western blot analysis and immunotrapping TEM, while the remainder of the tissue was used for the isolation of virus. Western blot analysis confirmed that the WT and the MBP TMV accumulated to similar levels in plants and that the MBP TMV coat protein was of a higher molecular weight than the WT, indicating successful insertion of the MBP (see **Supplementary Figure S1**). Immunotrapping TEM using antibodies against TMV illustrated that WT TMV could be successfully detected as distinct rods (300 nm length, 18 nm diameter) in plant material, and also that the MBP TMV existed as aggregated networks of particles of different lengths of up to 300 nm and diameters of 13–18 nm (see **Supplementary Figure S2**).

Subsequently the WT and MBP TMV particles were isolated from the remaining plant material and quantitated using spectrophotometry. It was found that both the WT and MBP TMV could be obtained to high and similar yields (960 mg/kg fresh leaf weight for WT TMV and 1120 mg/kg fresh leaf weight for MBP TMV). These findings are consistent with those reported by Turpen et al. (1995) who previously inserted various short 12–15 mer peptide sequences into this region and found that such modifications did not adversely affect accumulation of the virus in plants or impinge upon subsequent purification.

**FIGURE 1:**
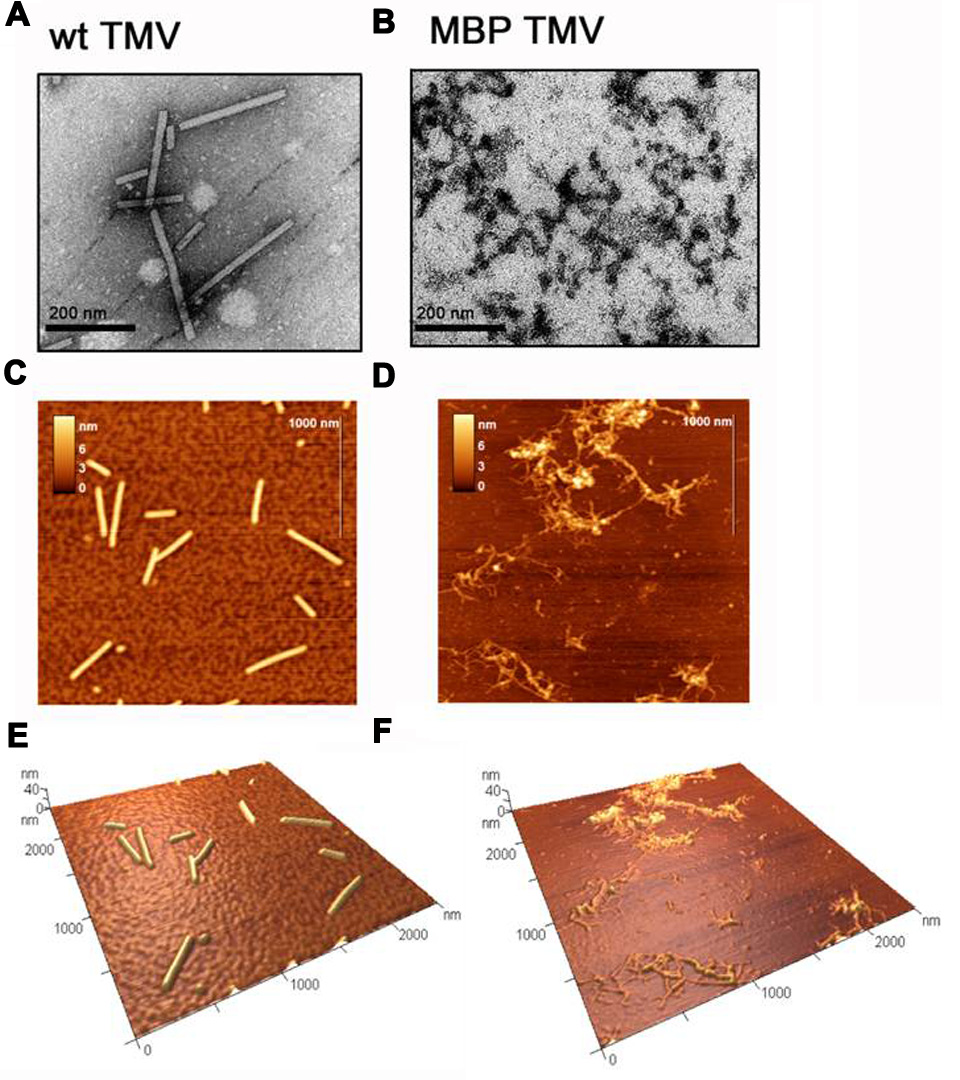
**Transmission electron microscope (TEM) images of **(A)** wild type (WT) tobacco mosaic virus (TMV) and **(B)** MBP TMV, with scale bars shown.** Atomic force microscopy (AFM) images with scale bars of **(C)** WT TMV and **(D)** MBP TMV, and their respective 3D projections **(E)** and **(F)**.

The integrity and morphologies of both TMV types was assessed using TEM (**Figures 1A,B**). As expected, it was found that the unmodified TMV predominantly existed as 300 nm length, 18 nm diameter rods (**Figure 1A**). In contrast the MBP TMV was found as a network of filamentous particles of various lengths of up to 300 nm and widths ranging from 13 to 18 nm (**Figure 1B**). The TEM data was found to correlate well with that obtained using atomic force microscopy (AFM; **Figures 1C–F**). AFM is a high-resolution technique that has been used in studies of biological macromolecules, in particular, for examination of mechanical properties and dimensions of virus particles (Dubrovin et al., 2007; Baclayon et al., 2010; Kuznetsov and McPherson, 2011; Dufrêne et al., 2013). It should be noted that physical measurements obtained using AFM usually give heights that are slightly reduced by compression and widths that are greatly increased by the effects of tip convolution (Keller, 1991). Since compression effects are much smaller than the effects of tip convolution, we present the height measurements. It was found using AFM that the heights of unmodified TMV particles were 13.2 ± 0.9 nm (**Figures 1C,E**), whereas those of MBP TMV were 5.9 ± 0.7 nm (**Figures 1D,F**). The smaller height of the MBP TMV structures may be due to improper assembly and/or instability, which might in consequence be more susceptible to AFM tip compression than the WT virus.

**FIGURE 2:**
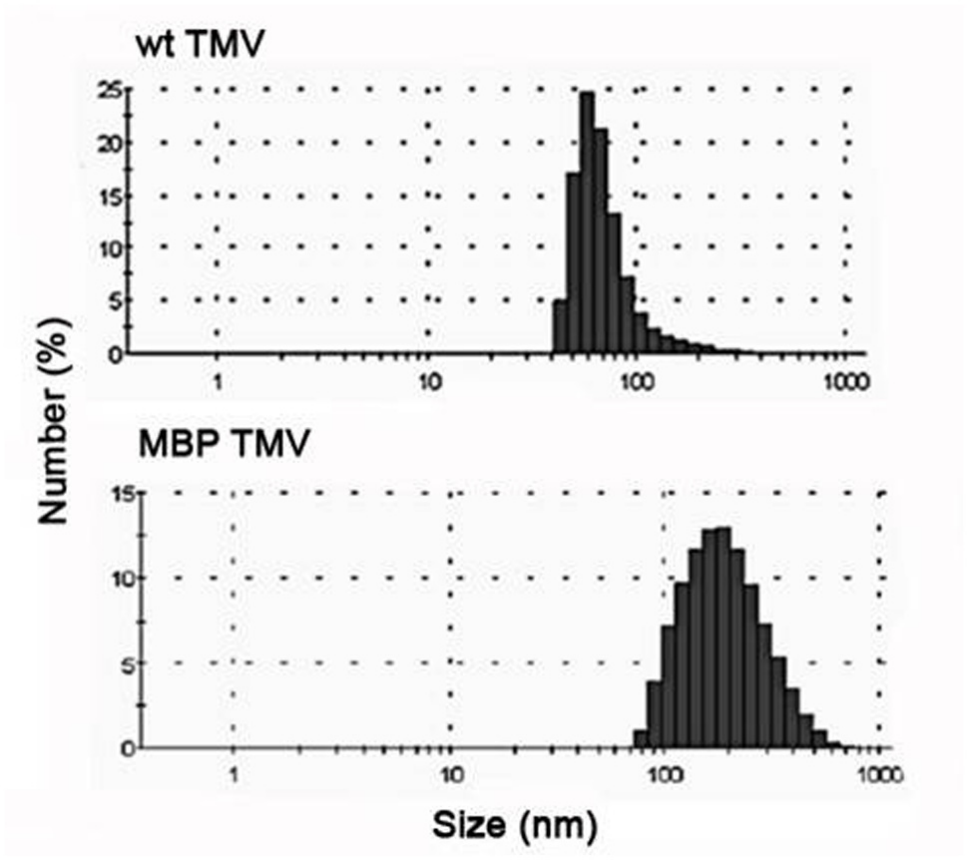
**Dynamic light scattering (DLS) analysis showing the size distributions of wt TMV and MBP TMV.** The size distributions reflect their aggregation state rather than the absolute hydrodynamic radius.

The aggregation observed for the MBP TMV sample was not an artifact of the microscope analysis since the DLS data suggested that MBP TMV has a distribution of larger particles than the WT TMV (**Figure 2**). Analysis of the hydrodynamic diameter of particles in solution by DLS showed that the unmodified TMV preparation had particles with an average size of about 70 nm, which in general coincides with the DLS data obtained for the other helical plant viruses (Nemykh et al., 2008; Makarov et al., 2013). In contrast, MBP TMV appeared as complexes with an average size of about 200 nm, which indicates that this virus is in an aggregated state in solution. Despite the fact that DLS is used primarily in the analysis of globular particles, it is also possible to use in the study of rod-shape viruses, especially where the aggregation states of the viruses are being considered rather than their absolute hydrodynamic radius.

### Production of Gold Nanoparticles using the MBP TMV and their Physicochemical Characterization

**FIGURE 3:**
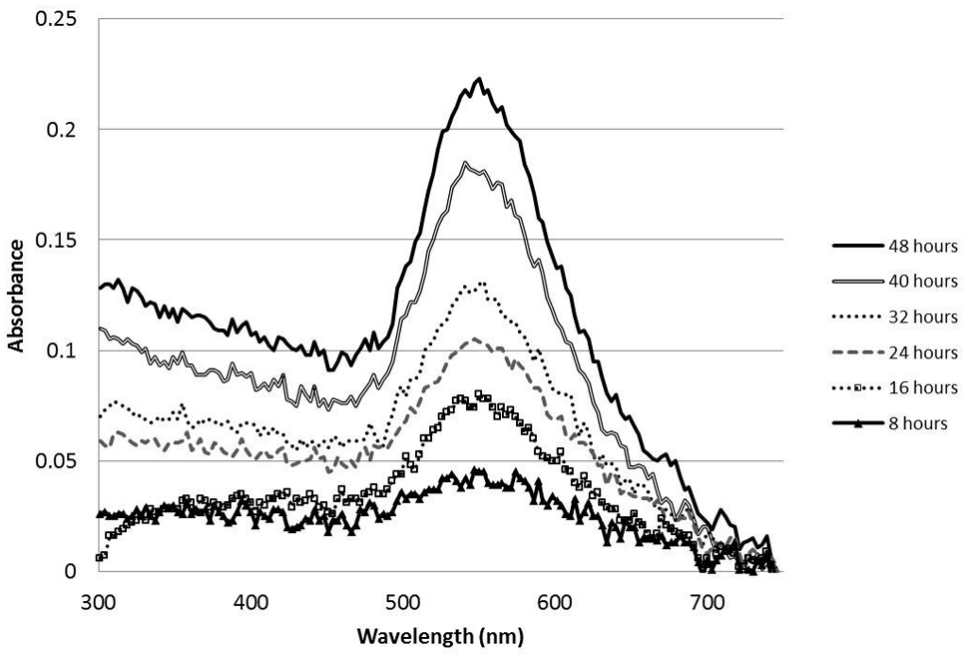
**UV-Visible spectral timecourse of gold nanoparticles formed from 200 μl reactions comprising 0.1 mg MBP TMV mixed and shaken at 100 rpm with 3 mM potassium tetrachloroaurate**.

In order to test the metal reducing capacity of the MBP TMV and unmodified TMV, 0.1 mg of each virus was added to 3 mM potassium tetrachloroaurate and shaken at 100 rpm. Within 32 h reactions containing the MBP TMV underwent a color change from yellow to pale violet; phenomena not observed with the WT TMV reactions. Given that these color changes are characteristic of nanoparticle formation, we analyzed the MBP TMV reactions over a period of 8 h intervals using UV-visible spectrophotometric methods in order to identify peaks indicative of nanoparticle production. UV-visible analysis showed that the MBP TMV reactions reached their conclusion within 48 h after reaction set up, producing a 500–600 nm absorption band with a peak at 550 nm which indicates gold nanoparticle formation (**Figure 3**; Singh et al., 2012). Such spectra were not observed with reactions containing WT TMV. Taken together these data indicate that MBP TMV can induce the formation of gold nanoparticles that are of a broad size range.

**FIGURE 4:**
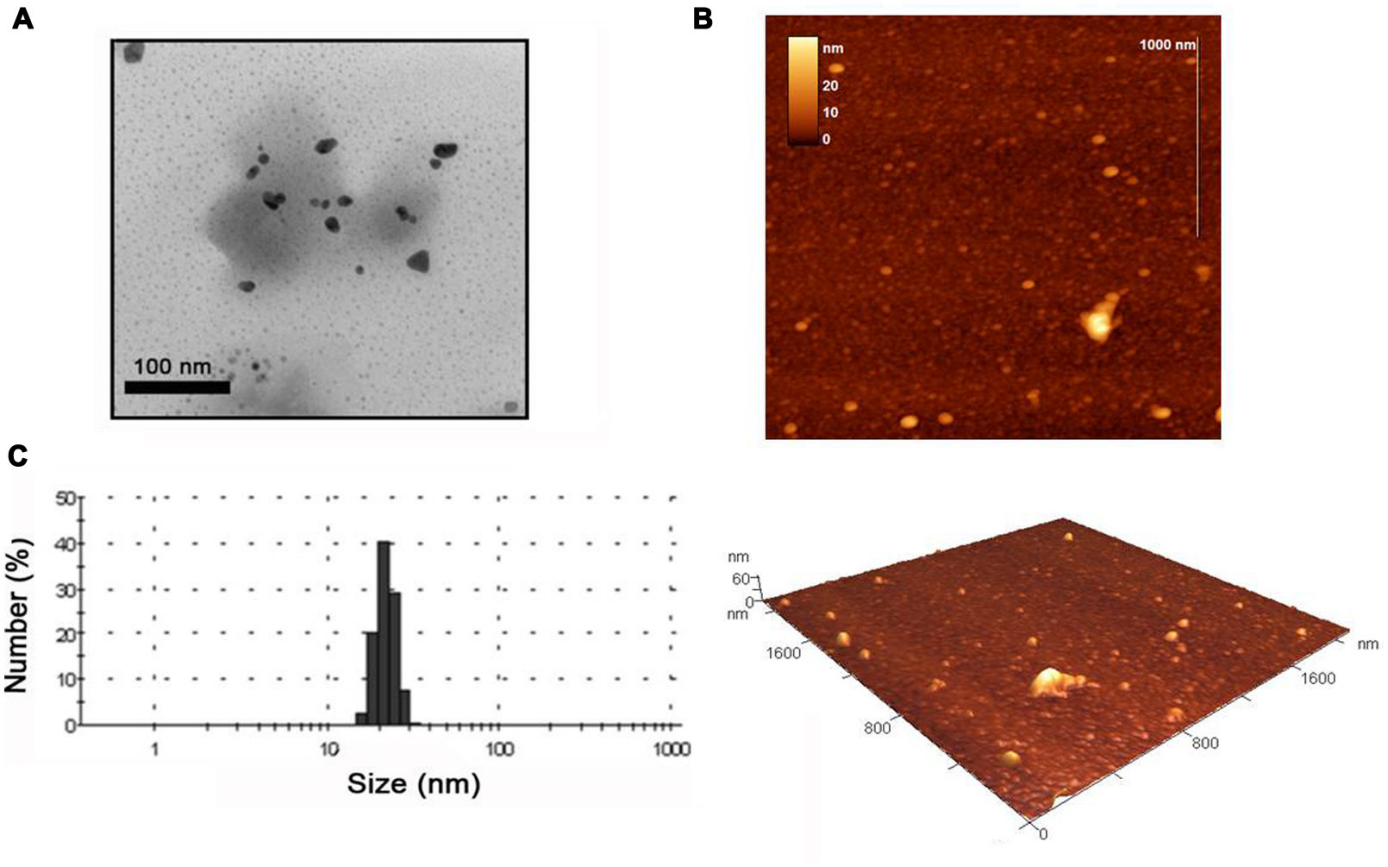
**Transmission electron microscope images of **(A)** gold nanoparticles produced using MBP TMV and 3 mM potassium tetrachloroaurate.** Scale bars are shown. **(B)** AFM images of the formed gold nanoparticles with appropriate scales. **(C)** DLS data showing size distribution of gold nanoparticles.

**FIGURE 5:**
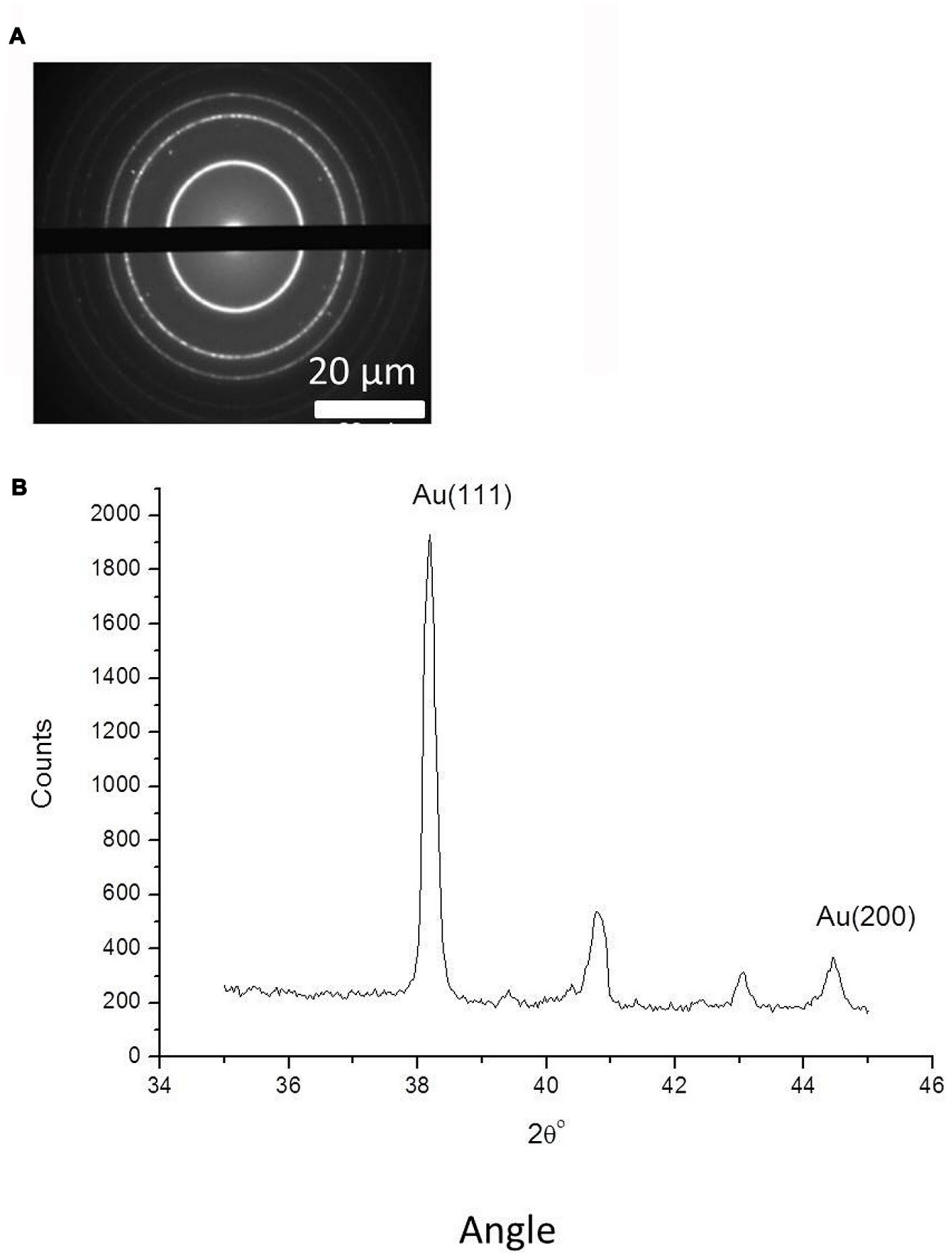
**(A)** Selected area electron diffraction analysis (SAED) analysis of gold nanoparticles, complete with scale bars. **(B)** Powder X-ray diffraction for gold nanoparticles measured in the 2Θ range from 35 to 45°, with indicated 111 and 200 peaks for metallic gold (JCPDS No. 04-0784).

We next analyzed the reactions using TEM and AFM and found that incubation of the WT TMV with the potassium tetrachloroaurate led to substantial aggregation of the virus with no obvious metallization or nanoparticle formation (see **Supplementary Figures 3A,C,E**), which is consistent with the lack of a color change in this reaction. In contrast the MBP TMV morphologies were unchanged by the incubation with gold salt (compare **Supplementary Figures 3B,D,F** with **Figures 1B,D,F**), and had some nanoparticles associated with the network. Interestingly, further TEM analysis revealed that for the MBP TMV reaction large numbers of unattached gold nanoparticles of 10–40 nm size ranges were clearly visible (**Figure 4A**), sizes which are consistent with the broad peaks observed in the UV-visible analysis. Using AFM the heights of the gold nanoparticles were in a size range of 9–33 nm (**Figure 4B**). The nanoparticle sizes visualized under the AFM and TEM also correlate well with the size measurements obtained using DLS approaches, which were 14–30 nm (**Figure 4C**). The samples were subsequently analyzed using SAED, which produced several diffraction ring patterns that depict different phase arrangements and illustrated the crystalline nature of the gold nanoparticles in the samples (**Figure 5A**).

Powder X-ray diffraction data in the 2Θ range from 35 to 45^o^ are shown in **Figure 5B**. The diffraction pattern has two peaks, corresponding to X-ray diffraction from the 111 and 200 planes of metallic gold (JCPDS No. 04-0784). This verifies the formation of gold nanoparticles with crystalline structure. The other minor peaks in **Figure 5B** can be attributed to X-ray diffraction from precipitated residual salts contained in the solution with the gold nanoparticles. We also measured EDX spectra of the separate gold nanoparticles to confirm their chemical composition and showed that their gold metal atom content was 71.92 ± 2%, with the remaining content predominantly comprising carbon.

An important consideration of nanoparticle formulations is their capacity to remain stable and not be prone to aggregation during storage; aspects which can greatly influence the physical and chemical properties and utility of the nanoparticles. Consequently, we measured the zeta potential of the formulations and found that the gold nanoparticles had a potential of -13 ± 0.2 mV, indicating that they have moderate stability and are not so prone to aggregation.

Thus, the MBP peptide sequence retains functionality when incorporated into surface exposed loops of the virus and is able to efficiently promote the synthesis of stable crystalline gold nanoparticles without the addition of exogenous chemical reducing agents.

## Discussion

In this paper we report that introduction of the MBP motif into a surface exposed region of the TMV CP can lead to the formation of thin networked virus structures that are quite distinct from the typical WT TMV rod morphology. However, this modified virus was not compromised in its ability to replicate and spread long distance and elicit the production of systemic symptoms. It has been reported that the CP of TMV is required for efficient long-distance movement through the plant. Previously amino acid changes have been introduced into key regions of the TMV CP that are required for appropriate self-assembly into the virus structure, and it was found that although some amino acid substitutions disrupted virion structures, they did not compromise virus movement and development of systemic symptoms (Bendahmane et al., 2007). This indicates that appropriate TMV CP assembly into rods is not always a prerequisite for long-distance spread and symptom development, which is consistent with our findings.

Previously Brown et al. (2000) identified that repetitions of amino acids such as SEKL and GASL could induce the slow formation of large gold nanoplates (500–1000 nm) when these peptides were mixed with gold salts. They also indicated that these sequences were neither covalently bonded nor incorporated into the gold crystal and thus may act as catalysts of nanoparticle formation. Later work by Tan et al. (2010) made the suggestion that flanking a pair tryptophans, which is a highly reducing amino acid, with the SEKL and GASL sequences (to give SEKLWWGASL) could further promote the speed of these reactions. This was the case, as reactions concluded at 4 h when a tryptophan pair was included versus the >20 h when they were not. Moreover inclusion of tryptophan pairs led to production of ∼40 nm diameter gold nanoparticles compared with the large >500 nm nanoplates when they were not. It was suggested that the reduction in size of the nanoparticle mediated by the WW was a consequence of increased reaction kinetics. In our paper we incorporated the SEKLWWGASL sequence into a surface exposed region of TMV, which led to the production of gold nanoparticles when mixed with potassium tetrachloroaurate. The reaction speeds we observed (48 h) were considerably slower than that reported for the gold nanoparticles synthesized using the SEKLWWGASL peptide (MBP; Tan et al., 2010). It is possible that the reduced speed in our MBP TMV gold reactions compared with that of the free MBP peptide used by Tan et al. (2010), might be due to slight differences in experimental setup or the effects of other sequences in the TMV surface which could further modulate reaction kinetics. Moreover these factors might also account for the small nanoparticles formed in spite of the slow reaction speeds that we observed.

It has previously been demonstrated that protein and organic acid mediated synthesis of nanoparticles often leads to direct incorporation of these organic components into the nanoparticle during nucleation and growth phases (Saptarshi et al., 2013; Makarov et al., 2014a,b; Sakulkhu et al., 2014), or in some cases they are not incorporated but rather weakly associate with the exterior of the nanoparticle (Brown et al., 2000; Tan et al., 2010). Both of these scenarios lead to decoration of the nanoparticle with charged groups which can result in particle repulsion; preventing aggregation and instability of the nanoparticles. In our experiments gold nanoparticles were not exclusively associated with the gross MBP virus networks, which may be consistent with the findings of Brown et al. (2000) who suggested that SEKL and GASL amino acid sequences can act as catalysts which do not become tightly bound to gold nanoparticles during or after their formation. The relatively stable zeta potentials and the carbon peak in the EDX analysis indicates that some organic component can associate with the gold nanoparticles, and if this is not the gross MBP TMV structures, it is possible that it might be subunits of these structures. For example the acidic gold solutions (pH < 3) we used in our experiments may have liberated some of the MBP TMV subunits from the gross structure and that these interact or become incorporated into the forming nanoparticles.

Traditionally WT TMV has been used for production of metallized virus structures. While unmodified TMV can actively bind metal ions via its surface exposed exterior OH and COOH groups, and also through its accessible OH and primary amine groups on the interior channel, these do not efficiently reduce the sequestered ions into metal nanoparticles or result in metallization of the virus (which is consistent with our data and that of other labs). For production of metallized TMV for a multitude of different applications (such as components in circuitry, batteries and fuel cells; Royston et al., 2008; Gerasopoulos et al., 2010), the metal ions sequestered by TMV have previously been reduced into metal via exposure to exogenous reducing chemicals (Shenton et al., 1999; Knez et al., 2004; Balci et al., 2006, 2012; Love et al., 2014). Interestingly it has previously been observed that the reducing conditions can be easily modulated to favor the deposition of very small ∼5 nm discrete gold metal nanoparticles on the surface of WT TMV, rather than complete surface metallization (Kobayashi et al., 2011, 2012). Moreover, these authors demonstrated that introduction of small metal binding peptides [such as gold (LKAHLPPSRLPS) and titanium binding sequences (RKLPDA)] into the TMV led to enhanced decoration of the virus surface with larger (∼10 nm) gold particles under these reducing conditions, which is in marked contrast to the WT TMV. It was suggested that such peptide sequences alters the charge balance on the TMV surface which in turn could promote nanoparticle nucleation in reducing conditions, forming partly metal decorated structures with applications in optical metamaterials (Kobayashi et al., 2011, 2012). In contrast to this work we carried out different modifications to the TMV CP and did not use exogenous reductants, but rather we relied upon the reductive capacity of the surface displayed peptide to produce nanoparticles. Unlike the reports by Kobayashi et al. (2011, 2012), in the reaction conditions we used there was not an obvious metallization of the TMV surface and moreover there was not a consistent association of the formed nanoparticles to the MBP TMV surface. A possibility for these differences is that in the other studies where exogenous reductants are used, the reactions are very fast (typically less than 2 h) and consequently the metal ions will be rapidly reduced *in situ* to form quickly expanding particles which may engulf TMV surface components and become firmly attached. Our reactions by comparison are much slower (48 h) and employ shaking, which may lower the capacity for nanoparticle engulfment of virus structures and enhance their liberation, especially considering that the peptides we surface displayed have been reported to very weakly associate with nanoparticles (Brown et al., 2000).

As an alternative approach to using reducing agents, Lim et al. (2010) modified TMV to surface display double cysteine residues which can direct metallization with palladium from palladium chloride at temperatures above 50°C; thus indicating that metallization of TMV is feasible in the absence of reducing chemicals. It is possible that investigating other parameters in our reactions such as increasing temperature and metal salt concentration and reducing shaking may lead to metallization of the MBP TMV, however, this remains to be tested.

In summary we carried out different modifications to the TMV CP and found that it did not become metallized, but rather it could generate free colloidal nanoparticles at room temperature in the absence of chemical reductants; which to our knowledge is the first report of a virus being used for this purpose.

A future challenge in this work will be to test different reaction conditions and virus modifications in order to: (1) broaden the types of metal nanoparticles that may be produced during this process, (2) improve the reaction kinetics and (3) develop new approaches for directed metallization of viruses under neutral, non-hazardous, room temperature conditions.

## Conflict of Interest Statement

The authors declare that the research was conducted in the absence of any commercial or financial relationships that could be construed as a potential conflict of interest.

## Supplementary Material

**FIGURE S1 |Western blot of plant material (lane 1), or plant material infected with wild type (WT) tobacco mosaic virus (TMV; lane 2) or MBP TMV (lane 3) probed with a TMV antibody.** Molecular weights are indicated on the right. It was found that the antibody did not react against uninfected leaf material (lane 1), however, a distinct band at the expected size of 17.5 kDa, representing the TMV CP was observed in WT TMV infected material (lane 2). In contrast, with the MBP TMV infected leaves, a slightly higher molecular weight band was observed, which is consistent with the net gain in weight brought about by the insertion of the MBP peptide (lane 3). The large rubisco subunit stained with ponceau is shown below the Western as a loading control.

**FIGURE S2 | Immunotrapping transmission electron microscope (TEM) was carried out on crude sap extracts of WT TMV **(A)** or MBP TMV **(B)** infected plants or uninfected plants **(C)**, using copper grids coated with antisera specific to TMV.** Scale bars are shown. The data indicates that this approach was successfully used to detect WT TMV rods **(A)** or network like structures of MBP TMV **(B)**. The lack of discernable structures in the uninfected plant sap extracts indicates the specificity of the antisera to TMV **(C)**.

**FIGURE S3 | Effect on the morphologies of WT TMV or MBP TMV due to incubation with 3 mM potassium tetrachloroaurate.** WT TMV and MBP TMV viewed under TEM (**A,B**, respectively), AFM (**C,D**, respectively) and AFM 3D projections (**E,F**, respectively). Scale bars are shown.
