# Supplementary figures and images for: A Genetically Modified Tobacco Mosaic Virus that can Produce Gold Nanoparticles from a Metal Salt Precursor
Source: Front Plant Sci. 2015 Nov 10;6:984. doi: 10.3389/fpls.2015.00984 (PMC4639705; doi:10.3389/fpls.2015.00984)

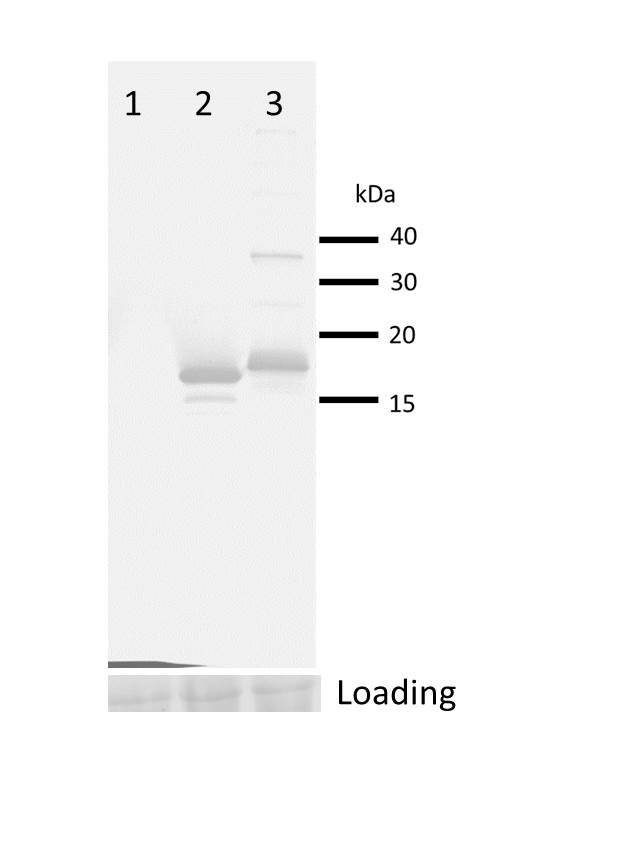

Supplement: Supplementary file 1 [file Image_1.JPEG]

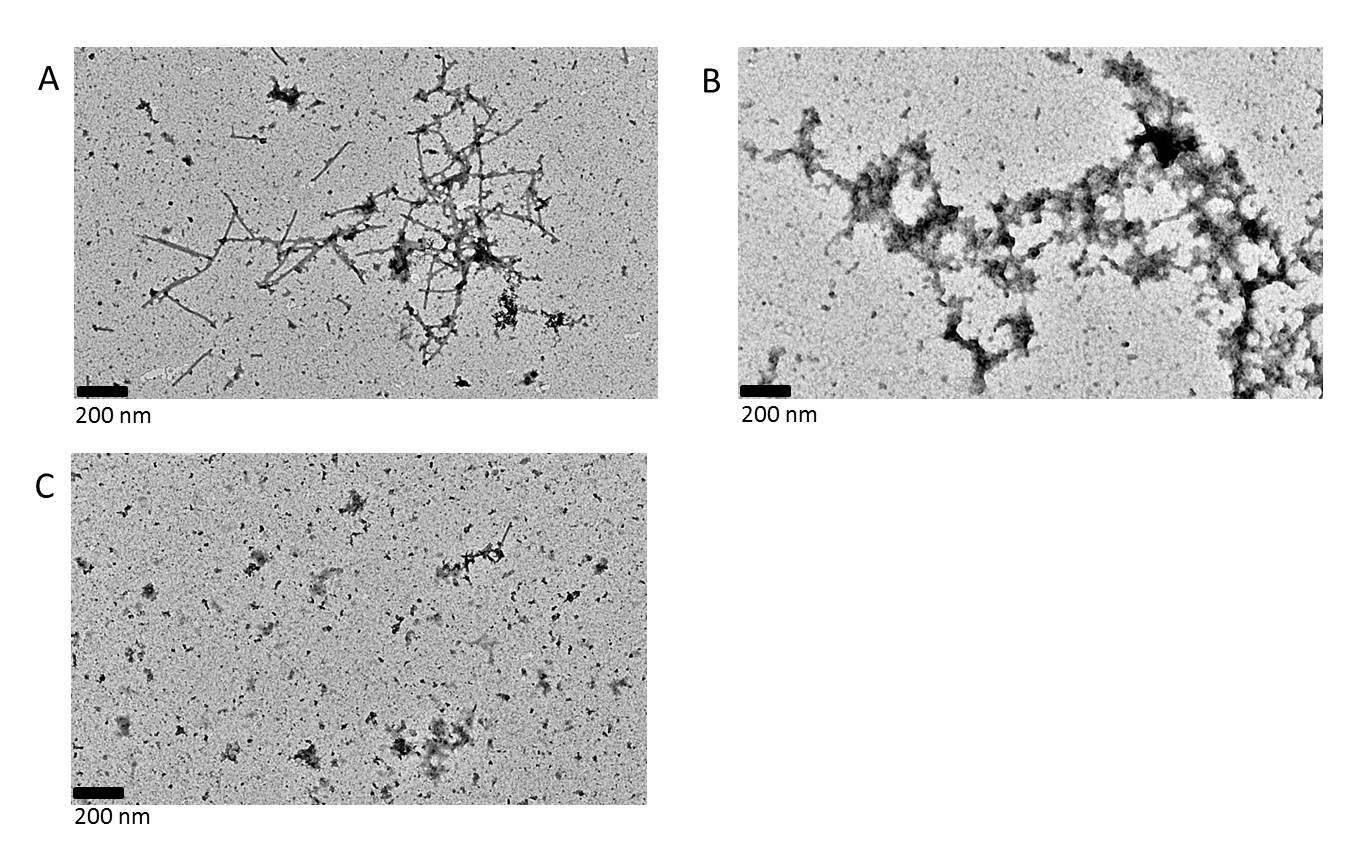

Supplement: Supplementary file 2 [file Image_2.JPEG]

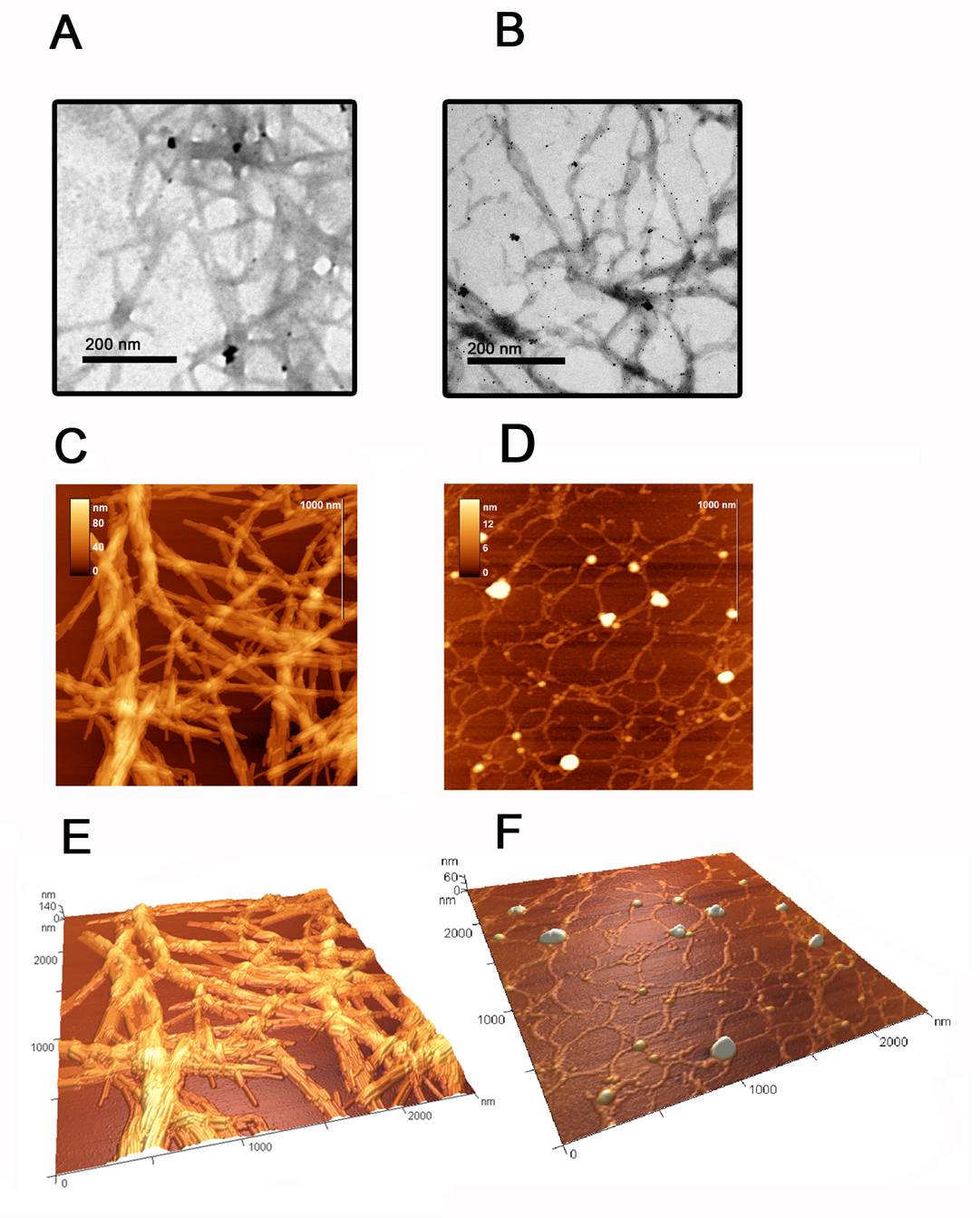

Supplement: Supplementary file 3 [file Image_3.JPEG]
